# The proportion of plant-based food consumption during midlife and cognitive health in later life in Australian women: data from the Women’s Healthy Ageing Project (WHAP)

**DOI:** 10.1007/s00394-025-03786-8

**Published:** 2025-10-09

**Authors:** Phuong Le, Cassandra Szoeke, Kaitlin Day, Russell Conduit, Sharayah Carter, Catherine Itsiopoulos

**Affiliations:** 1 School of Health and Biomedical Sciences, RMIT University, 124 La Trobe Street, Melbourne, VIC 3000 Australia; 2 Monash Centre for Health Research and Implementation, Monash University, Wellington Road, Clayton, VIC 3800 Australia; 3 Department of Nutritional Sciences, School of Life Course and Population Sciences, Faculty of Life Sciences and Medicine, King’s College London, London, UK; 4 Allied Health & Human Performance, University of South Australia, SA 5001 Adelaide, Australia

**Keywords:** Plant-based diet, Ageing adults, Female, Cognition, Alzheimer’s disease, Longitudinal study

## Abstract

**Purpose:**

Plant-based food (PBF) is well-known for its benefits for physical health; however, its impacts on brain health are less well understood, especially in ageing women. This study aimed to examine the association between different proportions of midlife daily PBF intake and late-life cognitive health among ageing Australian women.

**Methods:**

This study used data of 186 women who had dietary assessment at baseline (1998, aged 52–63) and cognitive assessments at follow-up (2012, aged 66–77) from the Women’s Healthy Ageing Project (WHAP). The cohort was divided into quartiles according to the proportions of PBF in their daily diet at baseline. Late-life cognitive function was assessed by Global cognitive composite score (GCCS)—a summary of z-scores of 13 cognitive tests of the Cogstate battery. Three regression models were conducted: unadjusted (N = 186), partially adjusted (age, education, energy intake; N = 186), and fully adjusted (age, education, energy intake, BMI, physical activity, smoking, APOE 4 allele status, N = 165).

**Results:**

In unadjusted and partially adjusted models (N = 186), women in the third quartile (Q3) (second highest consumption of PBF during midlife) had significantly higher GCCS in later life compared to those in the lowest quartile (Q1) (B = 0.39, 95% CI [0.13; 0.66]; *p* = 0.004 for the unadjusted model; B = 0.34; 95% CI [0.08; 0.61]; *p* = 0.012, for the partially adjusted model). However, this association was no longer significant in the fully adjusted model (N = 165) (B = 0.25; 95% CI [− 0.02; 0.51]; *p* = 0.07), where APOE 4 allele status emerged as a significant predictor (B = − 0.25, 95% CI [ − 0.45; − 0.04]; *p* = 0.02). This change may reflect the reduced statistical power due to smaller sample size and the confounding effect of the genetic risk factors. Among APOE 4 carriers, higher PBF quartiles (Q2–Q4) each predicted greater GCCS in unadjusted analyses; in the adjusted model, Q3 versus Q1 remained significant, but the overall model did not reach significance. Investigation into the change in PBF consumption from midlife to late-life revealed no association with late-life cognitive health.

**Conclusion:**

Midlife PBF consumption did not show a significant independent association with late-life cognitive health after fully adjustment for confounders in this cohort of older Australian women. However, these findings should be interpreted with caution as the small sample size and confounding factors might have affected the ability to detect a subtle effect of PBF on cognition. Future research is needed to explore this relationship in larger, more diverse samples and its complex interaction with genetic risk factors.

**Supplementary Information:**

The online version contains supplementary material available at 10.1007/s00394-025-03786-8.

## Introduction

### Background and rationale

Around 55 million people globally are currently suffering from dementia [1]. More than US$1.3 trillion worldwide was attributed to costs associated with dementia in 2019 and this figure is expected to rise to US$2.8 trillion by 2030 [1], and expected to further rise as the elderly population increases.

Currently, there are no effective treatments for dementia. One of the most challenging aspects of finding a cure for age-related cognitive diseases is their long prodrome. Thus, interventions that can be introduced during early stages of these diseases to delay or prevent them, i.e. during the patients’ middle age, are now the focus [2].

Women are twice as likely to develop Alzheimer’s Disease (AD) as men [3]. There are several factors that contribute to this but the change in hormones during menopause has been associated with the increased risk of dementia in women [4]. However, there has been only a limited number of studies that focus on women to design tailored interventions to prevent age-related cognitive diseases [2].

The effect of diet on human cognitive health has been demonstrated through various randomised controlled trials (RCTs) and observational studies (both longitudinal and cross-sectional studies) examining specific dietary patterns (DPs) such as the Mediterranean diet (MD), the DASH (dietary approach to stop hypertension) and the MIND (Mediterranean-DASH intervention for neurodegenerative delay) [5–8] diets. Higher adherence to these dietary patterns has been associated with better cognitive performance and less cognitive decline in older adults [5–8]. A common theme among these DPs is an emphasis on the high consumption of vegetables, fruits, nuts, and whole grains with limited consumption of animal products and processed foods.

Apart from these established DPs, in different cultures and regions, a variety of DPs can be drawn from the general population and the components of these DPs are inherently diverse. Thus, researchers have been studying the impact of DPs on cognition through researching a posteriori diets. An a posteriori diet is a dietary pattern derived from the participants’ eating habits by using a statistical analysis method such as factor analysis [9]. In some of these studies, the DPs that bring the most benefits to cognitive health also share some common characteristics with the established DPs namely a high emphasis on plant foods such as vegetables, legumes, fruit, and limited consumption of meat [10–12].

However, whether higher proportions of plant-based food (PBF) would equal greater benefits to cognition in the long term is still in debate. Studies looking at vegan diets have not been sufficient to support the beneficial effect of a plant-exclusive DP on cognitive health [13–15]. Several other studies supporting the beneficial effects of higher proportions of PBF on cognition have been those that looked at specific plant food groups such as fruits and vegetables [16–20]. There have also been studies using established indices measuring the quantity and quality of plant-based food such as the plant-based diet index and its impact on cognitive health, but the results have been inconsistent [21, 22] and did not demonstrate the direct relationship between total plant-based food consumption and cognitive health. Thus, this study aims to address this gap by looking at the proportion of plant-based food in a way that is inclusive of different diets and its direct link to cognitive health.

Considering the long prodromal nature of age-related cognitive diseases, longitudinal studies that span from mid-life to late-life are important in developing early effective interventions to prevent these diseases. However, evidence supporting the long-term impact of PBF on cognitive health has mainly been obtained through investigations of cohorts with the age at baseline older than 65 [10, 14, 15, 23, 24]. In addition, there has been evidence that suggests the change in estrogen level after menopause and several common menopausal symptoms have a strong correlation with age-related cognitive decline in women [25, 26] and the phytoestrogens and isoflavones in some plant foods have been shown to help manage these symptoms [27, 28]. Women are also more sensitive to developing AD than men if they carry the APOE 4 allele [29]. APOE 4 allele is the most important genetic risk factor associated with cognitive decline and AD [29, 30] thus studies focusing on women and APOE 4 status are critical. However, there have only been a few studies investigating the role of some dietary patterns that spanned from mid-life to late-life in a female-only cohort and these studies did not include a comprehensive cognitive battery assessment [31].

### Objectives

The main aim of this study is to explore the association between midlife consumption of PBF and late-life cognitive health in older women. This relationship was investigated by classifying different levels of PBF consumption during the participants’ middle age and observing whether these differences are correlated with participants’ different cognitive performances 14 years later.

## Methods

### Study design and participants

Data used in this study was from the Women’s Healthy Ageing Project (WHAP), which was initiated in 1990/1991. Participants were recruited in the Melbourne metropolitan area by random digit dialling with over 50,000 phone calls made. After eliminating unanswered calls and households with ineligible women, there were around 2800 women available for the study, and 2001 of those agreed to participate in the initial cross-sectional study of the WHAP [32]. Briefly, the cohort involved in the longitudinal study of the WHAP was 438 women, who have been followed up annually from 1992 to 1999 and intermittently since 2000 until now [32]. Participants’ entry age was from 45 to 55, before their menopausal transition. Data collected and details of the cohort have been previously described [32].

The University of Melbourne Human Research Ethics Committee approved the WHAP study with approval numbers listed as follows: 931149X (1992–1999); 010528 and 010411 (2002–2009); 1034765 and 1339373 (2012–2016); 1647448 (2017-current). The study followed the National Health and Medical Research Council Ethical Conduct in Human Research and the Helsinki Declaration, with informed consent provided by all participants before commencement.

### Variables

#### PBF intake

Data for plant-based food consumption of the participants was obtained from the Dietary Questionnaire for Epidemiological Studies Version 2 (DQES v2)—a validated food frequency questionnaire [33] that comprises 74 food items grouped into four categories: (1) cereal foods, sweets and snacks; (2) dairy products, meat and fish; (3) fruit and (4) vegetables (Supplementary Appendix 1). Portion sizes were estimated based on photographs included in the questionnaire. The intake frequency of each item ranges from ‘Never’ to ‘Three or more times per day’. Participants were asked to report their dietary intake during the previous year.

Participants’ intake of PBF was calculated by the summation of intake amount (g/day) of 54 plant and plant-derived food items. The chosen items for this study were all the crude plant-based items such as fruits and vegetables, and some minimally processed plant-based foods such as bread and rice (Supplementary Appendix 2). The PBF daily consumption percentage was calculated by dividing the total daily PBF intake by the total daily food intake (both in grams).

Changes in PBF intake from mid-life to late-life were calculated by subtracting participants’ data in 2012 from the corresponding data in 1998.

#### Cognitive assessment

From 2012, WHAP participants’ cognitive function was evaluated with a computerised cognitive testing battery—the Cogstate battery [34]. The battery comprises well-validated measures which are described in detail elsewhere [32]. Summary measures of five cognitive domains were covered, including: (1) memory (a composite score of 2 tests: CERAD delayed recall [35] and california verbal learning task (CVLT) delay [36]; (2) executive function (a composite score of 3 tests: Trail-making test B [37], Stroop C/D [38], and Tower of London [39]); (3) language (a composite score of 2 tests: Boston Naming test [40] and category fluency—animals test [41]); (4) processing speed (a composite score of Digit Symbol Coding test [42], Trail-making test A [37] and Letter-Number Sequencing test [42]); (5) visuo-spatial (a composite score of Rey-complex figure test [43], Judgement of Line Orientation [44] and Wechsler Adult Intelligence Scale III test [42]). Z-score of each test was calculated using mean and standard deviation from the raw score. The composite score for each cognitive domain was computed as the average of z-scores of the tests included. Then, these composite scores were averaged to form the global cognitive composite score (GCCS), and this score was used as the outcome to evaluate cognitive health at old age of the participants. Similar methodology to assess cognitive performance was utilised by Gardener et al. [6].

#### Covariates

Covariates in the adjusted models include participants’ age (in years), Body Mass Index (BMI) (kg/m^2^), level of education as a binary variable (≤ 12 years or > 12 years), current smoking status (yes or no), energy intake (kJ/day), physical activity (metabolic equivalent (MET) minutes per week) and APOE 4 allele carrier (yes or no). Physical activity was measured using the International Physical Activity Questionnaire (IPAQ) total score. APOE genotyping was carried out by restriction isotyping from the participants’ blood samples.

### Statistical methods

Statistical analyses were performed with IBM SPSS V.29 software. Visualisations of the results were obtained using SPSS and MS Excel.

Normal distribution was confirmed by visual inspection of histograms, Shapiro–Wilk normality tests and Q-Q plots prior to running any statistical analysis.

#### Study size

In 1998, 349 participants answered the DQES v2. At the 2012 follow-up, 187 of these participants had neuropsychological testing and were included in this study. One of these 187 participants was further excluded as their response to the DQES v2 survey was unreadable by the scanning machine. Thus, the main analyses in this study comprised 186 women. Comparisons between characteristics and outcome measures of the included and excluded participants were conducted by independent-samples t-tests and chi-squared tests for continuous and categorical variables, respectively. Results from the comparison are shown in Supplementary Table 1.

#### Primary analyses

Participants were divided into four equal quartiles, representing four levels of PBF proportion in their daily diet during midlife. Differences in characteristics between these four quartiles were quantified using one-way ANOVA and chi-squared tests for continuous and categorical variables, respectively.

The longitudinal association between quartiles of PBF consumption at baseline (1998) and the Global Cognitive Composite Score at follow-up (2012) was assessed by linear regression analysis. Three regression models were developed:

1. Unadjusted model with PBF consumption quartiles as the predictor for GCCS;
2. Partially adjusted model with age, education and energy intake as the covariates; this model was conducted on the full available sample of 186 participants; and.
3. Fully adjusted model with all the covariates—age, education, BMI, physical activity, smoking status, energy intake and APOE 4 allele status.

Participants with missing data on any of the covariates included in the fully adjusted model were handled using complete case analysis. This resulted in a sample of 165 participants for this model. Quartile 1 (lowest) of PBF consumption was the reference level for all models.

#### Secondary analyses

Secondary analysis regarding APOE 4 was conducted after the significance of APOE 4 allele status as a predictor was noted in the primary analysis. APOE 4 allele carriers and non-carriers were stratified to assess the influence of this genetic factor on the relationship between PBF consumption at midlife and cognitive health at late-life.

Linear regression model was conducted to assess the relationship between PBF consumption quartiles and Global Cognitive Composite Score in APOE 4( +) carriers, adjusted for relevant covariates, namely age, education and energy intake (excluding APOE 4 allele status).

The long-term impact of diets on late-life cognition of the participants was evaluated through the relationship between changes in PBF intake from baseline (1998) to follow-up (2012) and cognitive scores at follow-up (2012), using linear regression model. Cases exceeding ± 2 SD were identified as outliers in the model.

## Results

### Descriptive data

Characteristics of the participants at the 2012 cognitive assessment are presented in Table 1. Participants were stratified by the proportion of PBF in their daily diet at baseline (1998), which ranged from 19.6 to 78.7%. There were no significant differences in all criteria across the four quartiles of PBF intake (Table 1). It is worth noting that the four groups had an average BMI in the overweight range (25–29.9 kg/m^2^) (Table 1). The average daily energy intake of the participants, on the other hand, is lower than the average energy intake of the general female population in a survey conducted by the Australian Bureau of Statistics (ABS) during a similar timepoint—1995—which was around 6370 kJ to 8370 kJ [45].

**Table 1:** Participants stratified into four quartiles based on the percentage of plant-based food in their midlife daily diet and their characteristics (N = 186)

Levels of PBF intake	Year of measurement	Quartile 1 (19.6–44.5%)	Quartile 2 (44.5–52%)	Quartile 3 (52–59.4%)	Quartile 4 (59.6–78.7%)	*p*-value
Criteria						
N		**46**	**47**	**47**	**46**	
Age (yrs)	**2012**	**70.4**	**69.5**	**70.1**	**70.1**	0.39
Mean (sd; range)		(2.9; 66.1–77.3)	(2.3; 66.2–74.1)	(2.6; 66.2–75.9)	(2.5; 66.3–77.1)	
BMI (kg/m^2^)	**2012**	**29.2**^**b**^	**28**	27.6^**a**^	**26.9**^**b**^	0.25
Mean (sd; range)		(5.1; 18.3–42)	(4.6; 19.5–40.5)	(6.7; 19.4–54.2)	(5.1; 18.8–40)	
Education	**2012**					0.09
≤ 12 years, n (%)		**33** (71.7%)	**22 ** (46.8%)	**24**(51.1%)	**26** (56.5%)	
> 12 years, n (%)		**13** (28.3%)	**25 ** (53.2%)	**23** (48.9%)	**20** (43.5%)	
Currently smoking n (%)	**2012**	**5 **(10.9%)	**4** (8.5%)	**3**^c^ (6.4%)	**2** (4.3%)	0.71
APOE 4 carrier n (%)	**N/A**	**12**^c^ (26.1%)	**9**^a^ (19.1%)	**17**^a^ (36.2%)	**11**^e^ (23.9%)	0.32
Physical activity (METmin/wk) Mean (sd; range)	**2012**	**3918. 9**^d^ (2825.9; 618–11,700)	**5640.2** (6042.8; 206–31,404)	**4237**^d^ (3188.2; 30–12,915)	**5775.6** (6054.9; 180–28,282.5)	0.17
Energy intake (kJ/day) Mean (sd; range)	**1998**	**5998.3** (3184.4; 3271.2–23,579.4)	**5739.8** (1476; 2776.9–10,220)	**5799.1** (1877.6; 2495.6–13,406.5)	**5607.1** (2021.8; 2872.3–12,164.3)	0.86
Alcohol consumption (g/day) Mean (sd; range)	**1998**	**159.7** (176.4; 0–725.5)	**170.5** (248.9; 0–1402.5)	**159.1** (201.3; 0–1154.2)	**159.5** (233.5; 0–1319.1)	0.99
Total food intake (g/day) Mean (sd; range)	**1998**	**1135.9** (392.6; 513.8–2679.4)	**1168.6** (307.5; 645–1810.1)	**1215.6** (377.7; 642.5–2162.4)	**1212.3** (363.9; 538.5–1990.9)	0.67

^a^N = 46, ^b^N = 45, ^c^N = 44, ^d^N = 43, ^e^N = 42

### Association between midlife PBF consumption (1998) and late-life GCCS (2012)

**Fig. 1:**
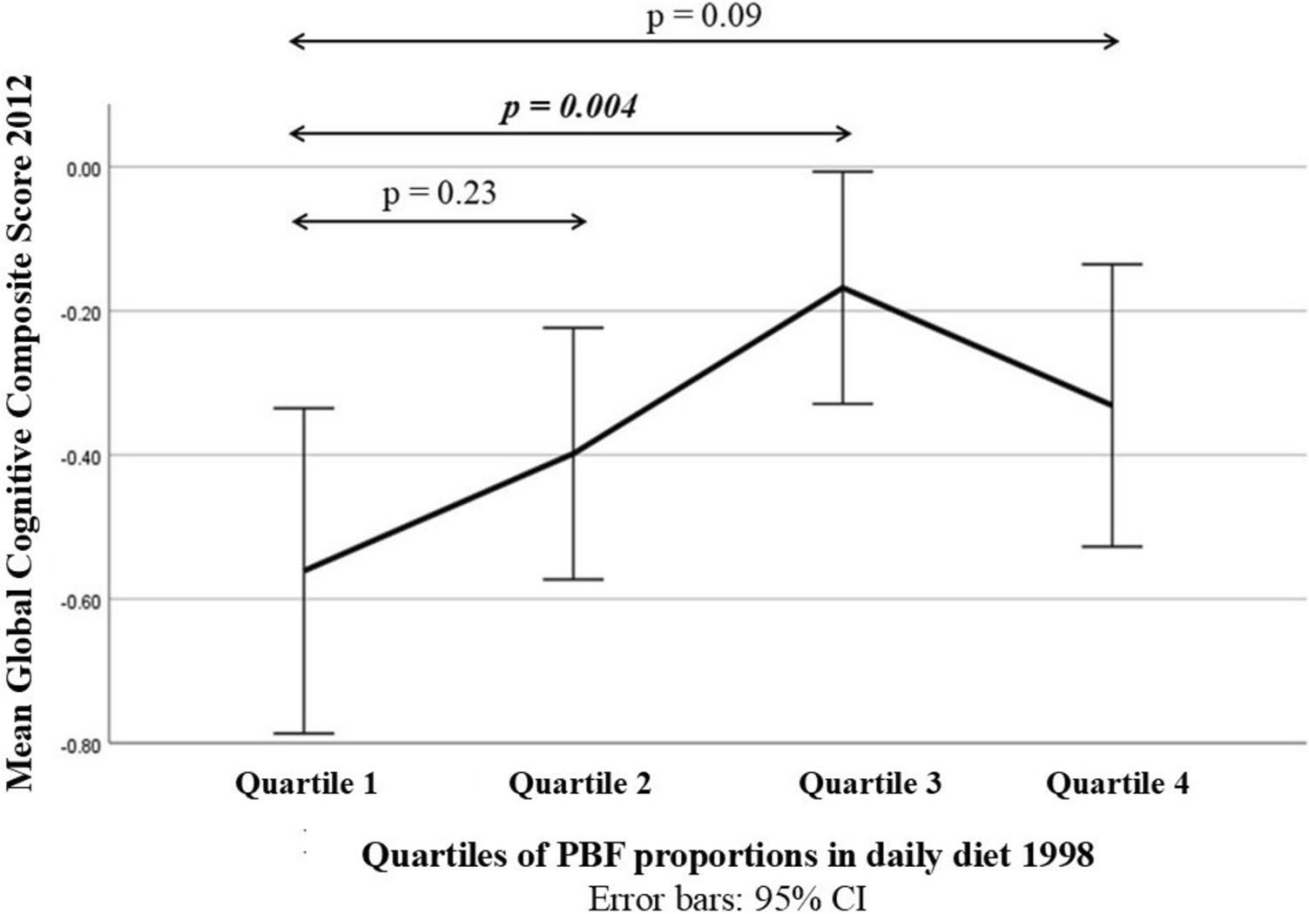
Line chart comparing means of Global Cognitive Composite Score (2012) and their 95% confidence intervals between the four quartiles of Plant-based food proportion in the participants’ daily diet (1998). Quartile 1’s mean score = − 0.56; Quartile 2’s mean score = − 0.4; Quartile 3’s mean score = − 0.17; and Quartile 4’s mean score = − 0.33 (*p* = 0.034)

The mean GCCS at late-life (2012) of the four quartiles of PBF proportion at midlife (1998) is illustrated in Fig. 1. Quartile 1 (Q1) had the lowest mean score, while Quartile 3 (Q3) had the highest score, and the difference between these two quartiles was significant (*p* = 0.004). It is noteworthy that the mean GCCS had an upward trend from Q1 to Q3, then followed by a slight decline in Q4 (Fig. 1).

Table 2 shows results of the unadjusted and the two adjusted linear regression models for the longitudinal association between PBF intake quartiles and GCCS. In the unadjusted model (N = 186), PBF proportion was a significant predictor of GCCS (*p* = 0.034). Specifically, Q3 had significantly higher GCCS compared to those in Q1 (B = 0.39, 95% CI [0.13; 0.66], *p* = 0.004) (Table 2). No other PBF quartiles showed a significant difference from the reference group.

**Table 2:** Linear regression models—unadjusted and adjusted—for the association between midlife plant-based food proportion (1998) and late-life global cognitive composite score (2012)

Variable	Unadjusted model	Partially adjusted model (Age, education, and energy Intake)	Fully adjusted model (Age, education, energy Intake, BMI, smoking status, physical activity and APOE 4 status)
	Beta (95% CI); *p*-value	Beta (95% CI); *p*-value	Beta (95% CI); *p*-value
Sample size	**N = 186**	**N = 186**	**N = 165**
*PBF proportion quartiles*			
Quartile 1	Reference	Reference	Reference
Quartile 2	0.16 (− 0.1; 0.43); 0.23	0.098 (− 0.17; 0.37); 0.47	− 0.09 (− 0.34; 0.17); 0.5
Quartile 3	0.39 (0.13; 0.66); ***0.004***	0.34 (0.08; 0.61); ***0.012***	0.25 (− 0.02; 0.51); 0.07
Quartile 4	0.23 (− 0.04; 0.5); 0.09	0.19 (− 0.07; 0.46); 0.15	0.03 (− 0.24; 0.29); 0.85
*Covariates*			
Age (years)		− 0.01 (− 0.04; 0.03); 0.78	0.01 (− 0.02; 0.05); 0.49
Education (≥ vs. < 12 years)		0.24 (0.05; 0.44); ***0.013***	0.16 (− 0.03; 0.35); 0.09
Energy intake (kJ/day)		5.09e-6 (0; 0); 0.81	2.69e-6 (0; 0); 0.92
BMI (kg/m2)			− 0.01 (− 0.03; 0.01); 0.28
Smoking status (yes vs. no)			− 0.03 (− 0.38; 0.33); 0.89
Physical activity (METmin/week)			1.14e-5 (0; 0); 0.25
APOE 4 status (yes vs. no)			− 0.25 (− 0.45; − 0.04); ***0.02***
*Model’s significance* (*p-value)*	***0.034***	***0.017***	0.059
*Adjusted* R^2^ *value*	0.031	0.051	0.048

The purpose of the bold sample size, and italic text is to enhance readability for readers

The bolditalic p-value's is intended to tell that it is statistically significant, compared with normal text p-value's

In the partially adjusted model (N = 186) which included age, education, and energy intake as covariates, the overall association remained significant (*p* = 0.017). The significant difference between Q1 and Q3 of PBF consumption persisted (B = 0.34, 95% CI [0.08; 0.61]; *p* = 0.012). Additionally, education also became a significant predictor of GCCS (B = 0.24, 95% CI [0.05; 0.44]; *p* = 0.013) (Table 2).

In the fully adjusted model (with age, education, BMI, energy intake, physical activity, smoking status and APOE 4 status), the sample size was reduced to 165 participants due to complete case handling of missing data across several covariates, namely BMI, physical activity, smoking status and APOE 4 status. This model was marginally insignificant with p = 0.059 (Table 2). The contrast between PBF Q3 and Q1 was no longer significant (B = 0.25, 95% CI [− 0.02; 0.51], *p* = 0.07). Notably, APOE 4 status emerged as a significant predictor of GCCS (B = − 0.25, 95% CI [− 0.45; − 0.04], *p* = 0.02) (Table 2).

### Effect of APOE 4 allele status in the association between midlife PBF consumption (1998) and late-life GCCS (2012)

**Fig. 2:**
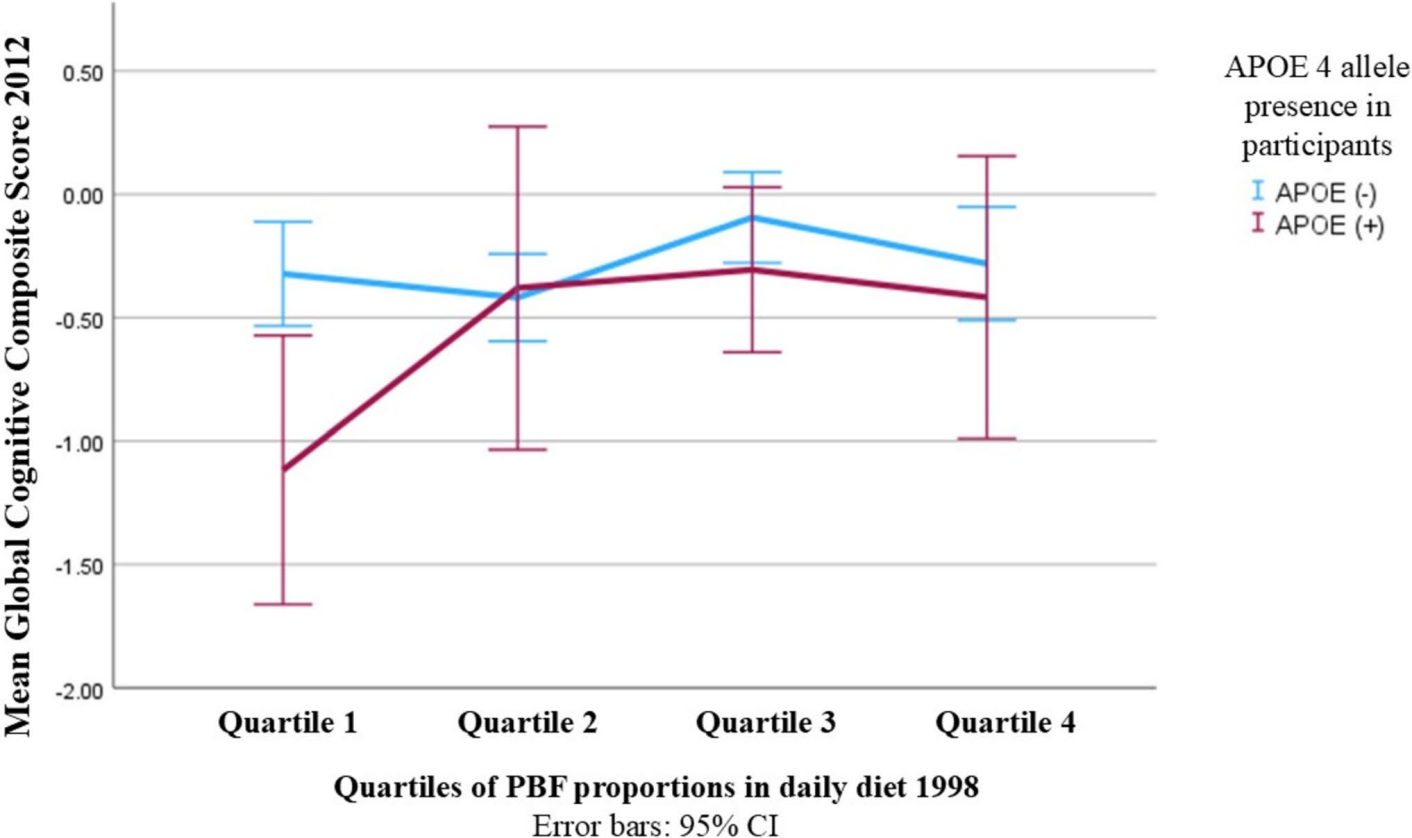
Line chart of Global Cognitive Composite Score (2012) and their 95% confidence intervals between the four quartiles of Plant-based food proportion in APOE 4( +) and APOE 4(−) participants. In APOE 4(−) group (N = 129), Quartile 1’s mean score = − 0.32; Quartile 2’s mean score = − 0.42; Quartile 3’s mean score = − 0.09; and Quartile 4’s mean score = − 0.28 (* p* = 0.137). In APOE 4( +) goup (N = 49), Quartile 1’s mean score = − 1.12; Quartile 2’s mean score = − 0.38; Quartile 3’s mean score = − 0.31; Quartile 4’s mean score = − 0.42 (*p* = *0.047*)

As APOE 4 status was a significant predictor in the fully adjusted linear regression model (Table 2), the cohort was stratified by the presence of APOE 4 allele for further investigation. There were 129 participants who were APOE 4 non-carriers (APOE 4(−)) and 49 APOE 4 carriers (APOE 4( +)). Mean GCCS across the four quartiles of PBF proportion in APOE 4( +) and APOE 4(−) groups were illustrated separately in Fig. 2. Differences between the four means in APOE 4 non-carriers were not significant (*p* = 0.137) but those in APOE 4 carriers were significant (*p* = 0.047) (Fig. 2).

In the unadjusted model, midlife PBF consumption significantly predicted late-life GCCS within this sub-cohort (Table 3) (*p* = 0.047). Specifically, all quartiles of PBF proportion had significantly higher GCCS than Quartile 1 in these participants. As the sample size for this group was rather small, N = 49, only a limited number of covariates could be included in adjusted models to maintain statistical power. Therefore, only the partially adjusted model with age, education and energy intake as covariates was conducted for this group. This model did not achieve overall statistical significance to predict GCCS (p = 0.16); however, it is worth noting that the difference between Q1 and Q3 remained statistically significant (B = 0.67, 95% CI [0.02; 1.32]; *p* = 0.04).

**Table 3:** Linear regression models—unadjusted and adjusted—for the association between midlife plant-based food proportion (1998) and global cognitive composite score (2012) in APOE 4(+) participants

Variable	Unadjusted model	Partially adjusted model (Age, Education, and Energy Intake)
	Beta (95% CI); *p*-value	Beta (95% CI); *p*-value
Sample size	**N = 49**	**N = 49**
*PBF proportion quartiles*		
Quartile 1	reference	reference
Quartile 2	0.74 (0.04; 1.44); ***0.04***	0.53 (− 0.26; 1.32); 0.18
Quartile 3	0.81 (0.21; 1.41); ***0.009***	0.67 (0.02; 1.32); ***0.04***
Quartile 4	0.70 (0.04; 1.36); ***0.04***	0.57 (− 0.16; 1.3); 0.12
*Covariates*		
Age (years)		− 0.02 (− 0.12; 0.08); 0.68
Education (≥ vs. < 12 years)		0.30 (− 0.24; 0.84); 0.27
Energy Intake (kJ/day)		− 9.82e-6 (0; 0); 0.86
Model’s significance (*p*-value)	***0.047***	0.16
Adjusted R^2^ value	0.104	0.08

The purpose of bold sample size and italic text is to enhance readability to readers

The bolditalic p-value's is to indicate that it is statistically significant compared with normal-text p-value's

More detailed investigation was conducted where other covariates from the full set of covariates were adjusted in this sub-cohort to see if the variance in GCCS could be better explained (Supplementary table 2). Consistent insignificant results were found in these models, indicating a lack of strong predictor for GCCS in this sub-cohort (Supplementary table 2).

### Association between change in PBF consumption from midlife (1998) to late-life (2012) and GCCS at late-life (2012)

Dietary data for 167 of 186 participants were available in 2012 to conduct this analysis. Overall, the cohort investigated had a decrease in PBF proportion in their daily diet from midlife (1998) to late-life (2012) (Supplementary table 3). Simple linear regression analysis was conducted to investigate the relationship between this change in percentage of PBF and GCCS at late-life (2012) in these participants. No statistically significant association was found (B = − 0.01, 95% CI [− 0.82; 0.8]; *p* = 0.98) (Supplementary Table 4). Extra analysis was done to ensure outliers did not influence the results. Casewise diagnostics identified 7 outliers at ± 2 SD, so these 7 cases were excluded. However, the relationship between change in PBF proportion over 14 years and GCCS was still found to be non-significant (Supplementary table 4).

## Discussion

This study investigated the longitudinal association between plant-based food (PBF) proportion consumed at midlife and cognitive performance in later life. In unadjusted and partially adjusted models, higher PBF proportions in daily diet at midlife was associated with greater GCCS at late-life. However, after full adjustment, the PBF-cognition association was no longer statistically significant. Below, we discuss plausible reasons and contextualise our findings.

### Midlife PBF consumption (1998) and late-life cognitive health (2012)

In the unadjusted model and partially adjusted model where the full sample size (N = 186) was utilised, there was a statistically significant association between midlife PBF proportion and late-life GCCS. In both of these models, individuals in the third quartile (Q3) of PBF consumption showed significantly higher GCCS than those in the lowest quartile (Q1). The consistent significant contrast between PBF Q3, but not Q4 (highest PBF consumption group) and Q1 in these models suggests a possible non-linear relationship between PBF and cognition. In fact, other studies have also reported similar results. For example, a study by Morris et al. [18] reported that cognitive decline was slower in the fourth quintile (2.8 servings/day) than in the fifth quintile (4.1 servings/day) of vegetable consumption. A study by Kang et al. [17] investigated an all-female cohort at older age and also reported that the most significant differences in the participants’ cognitive scores was observed at the fourth quintile of cruciferous vegetables consumption. Interestingly, in the partially adjusted model, education also emerged as a significant predictor of GCCS, which is consistent with findings from existing literature regarding the important role of education in maintaining cognitive health in older adults. In multiple studies conducted by Stern and colleagues [46–49], people with higher education levels were less likely to develop AD when they reach old age.

However, in the fully adjusted model, the PBF-cognition relationship attenuated (*p* = 0.059, Q1-Q3 contrast, *p* = 0.07) (Table 2)**.** This change is likely attributable to several factors. First, the statistical power of this model has been reduced due to the reduction of sample size. The changes in effect sizes and significance level from the models with full sample size to those of this model have reflected the strong impact of small sample size on the detectability of the true effect of PBF on cognition. Previous review articles have also reported similar limitation of longitudinal cohort studies when investigating the long-term impact of diets [14, 50]. Another factor that could have affected the change in this PBF-cognition association was the confounding effect of APOE 4 allele status. Adjusting for APOE 4, a well-established genetic risk factor for cognitive decline, rendered the association between PBF consumption and cognition non-significant, suggesting that initial models likely suffered from residual confounding.

Some previous observational studies also did not find a significant association between adherence to a plant-based diet and cognitive health in older adults [21, 51–53]. In our study, the weak association between PBF and cognition can be explained by several reasons. First, the plant foods in this study accounted for the total plant-based food actually consumed by the participants, so the presence of unhealthy PBF (e.g. tinned fruits) might have masked some beneficial effects of the healthy ones (e.g. fresh fruits and vegetables) [51, 54]. These findings warrant the importance of investigations into dietary quality versus quantity for developing appropriate interventions for specific population needs, especially in a high-risk population like ageing women. In addition, the non-linear relationship between PBF and cognition where the highest quartile of PBF consumption (Q4) had lower cognitive score than the third quartile, might suggest a possible optimal proportion of PBF to cognitive health. Even though the diet of those in Q4 was not plant-based exclusively, i.e. a vegan diet, they could be facing the lower availability of several essential nutrients that are necessary for supporting brain health such as vitamin B12 which we primarily obtain from animal products [13]. Vitamin B12 is an essential vitamin in controlling homocysteine levels, which is an important factor for better cognitive performance [55].

In addition, literature has shown mixed results regarding which cognitive domains are most impacted by plant-predominant dietary patterns [10, 56] so this study where only global cognitive function was analysed might have obscured the effect of PBF on certain cognitive domains. Several research studies have found that different plant foods and phytochemicals present in those foods might affect each cognitive domain at different levels [15, 57]. In addition, in post-menopausal women, not only the change in estrogen level but also the vasomotor symptoms (VMS) such as hot flashes and night sweats have been shown to have a strong impact on brain health and changes in cognitive performance at old age [25, 26]. Plant foods containing phytoestrogens and isoflavones such as soy, have been reported to increase estradiol and confer beneficial effects in managing VMS [27, 28], which in turn, might help in protecting cognitive health in post-menopausal women. Thus, further investigations into these specific plant foods could greatly contribute to the understanding of how a woman’s brain ages and developing tailored interventions for this specific population.

### Effect of APOE 4 allele on the relationship between midlife PBF consumption and late-life cognitive health

Since APOE 4 was significant in the fully adjusted model (B = − 0.25, 95% CI [− 0.45; − 0.04]; *p* = 0.02) (Table 2), we stratified the cohort by carrier status. The impact of PBF consumption was only found significant in the APOE 4(+) group (Fig. 2). This might be attributed to the fact that APOE 4 allele, compared with its isoforms (APOE 2 and 3), is a less effective cholesterol transporter, which results in elevated plasma cholesterol and low-density lipoprotein and is involved in the dysregulation of cholesterol homeostasis [58, 59]. As a results, lipid-related pathways involved in neuronal impair mechanisms are affected in APOE 4 carriers, making them more sensitive to dietary lipid [58, 60]. Higher PBF consumption, thus, could exert a more pronounced benefit in these individuals as it is generally lower in saturated fat and dietary cholesterol, which helps modulating cholesterol levels and improving lipid profiles in APOE 4 carriers [60]. Gardener et al. [6] also reported similar findings where they found more significant association between adherence to a Mediterranean diet with better cognitive performance in APOE 4 carriers than in non-carriers. Despite the insignificance of the model adjusted with age, education and energy intake, the contrast between PBF Q3 and Q1 on GCCS remained statistically significant (B = 0.67, 95% CI [0.02; 1.32]; *p* = 0.04). This persisted association, even with limited statistical power of a small sample size (N = 49), further supports the more pronounced benefits that PBF bring to APOE 4 carriers. These results may help the development of interventions tailored to APOE 4 carriers, especially women carriers, because women with APOE 4 allele are believed to be more likely to develop AD than their male counterparts due to greater hypometabolism and brain atrophy [30].

### Change in PBF consumption from midlife to late-life and late-life cognitive health

Changes in the participants’ PBF proportions might somehow reflect the healthiness of the participants’ diet over time. In this cohort of the WHAP, participants showed an overall decrease in the consumption of PBF over the period of 14 years. The change in this cohort’s diet has been discussed in more details in a study by Hill and colleagues [61]. Their study examined the changes in nutrition in the WHAP cohort for the same period of time (1998–2012). It was shown that the participants had shifted towards a poorer diet, which was demonstrated by changes in several indices such as an increase in Dietary Inflammatory Index (DII), decrease in MD adherence score, and increase in energy built by fat [61]. Hill and colleagues postulated possible reasons for this change in the WHAP cohort diet could have been changes in age-related energy requirements, marriage status and social engagement of the participants.

This present study did not find any association between the change in PBF proportion over 14 years and late-life cognition in these participants. There could be due to several reasons. First, how the shift in their diet had progressed over this timeframe was unknown due to the lack of in-between assessments of the participants’ dietary habits. Thus, whether the change in their diet had happened close to the follow-up timepoint or close to the baseline timepoint was not clear, so the long-term impact of the diet might have been obscured. It should also be acknowledged that the cognitive outcomes might have been impacted by other unmeasured factors in this analysis such as the overall diet quality or the baseline cognitive performance of the participants.

### Strengths and limitations

Strengths of this study included the use of longitudinal data of an all-female cohort from the WHAP study with validated measures for dietary assessment and cognitive assessment. The long follow-up time of the same cohort of women transitioning from middle to old age enabled the investigation of the impact of certain risk factors on cognition at late-life, addressing the long prodromal nature of age-related cognitive health issues. However, as this study focuses on women of this age frame, the findings could not be generalised towards other age groups of Australian women. As dietary habits tend to change over time, it was also difficult to compare the dietary habits of this cohort with the general population of the same age during the same time period. Comparing to a dietary survey in 1995 by the Australian Bureau of Statistics (ABS), this study’s participants had a slightly higher fruits and vegetables (FV) intake and a lower cereals intake than the female group surveyed [45]. However, the national survey included data for all female from 18 years of age and above while this cohort was 52 to 63 and were mostly Caucasian. Nevertheless, the wide range of PBF proportion in the investigated cohort enables a broad comparison between the different levels of PBF consumption.

This study is subject to several important limitations, the primary of which is the small sample size. The reduction of the sample size from 186 to 165 in the fully adjusted model significantly limited our statistical power, affecting the ability to detect a potential but subtle effect of PBF consumption on cognitive health. Furthermore, complete case analysis due to missing data might have introduced selection bias. The non-significant findings in the adjusted models should thus be interpreted with caution, as a larger sample size might yield different results.

This study is also subject to common limitations associated with studies of diet and self-reported questionnaires. Some food intakes might have been underestimated or overestimated. In addition, the dietary questionnaire could not possibly include all PBF that each participant had consumed. Thus, it might have missed some PBF that have been proven important in supporting cognitive health such as coffee and olive oil [8, 62], which potentially leaves a gap in this investigation. Another limitation of this study was that the socio-economic factor of the participants was not included in the analysis. People from different socio-economic statuses might show substantial differences in their choice of food due to different accessibility and economic freedom [63].

## Conclusion

This study has investigated the association between midlife plant-based food consumption and late-life cognition in a cohort of community-dwelling Australian women. In this cohort, midlife PBF proportion was associated with better late-life cognitive performance in unadjusted and partially adjusted models, but this association attenuated (*p* = 0.059) after full adjustment for confounders, especially APOE 4 status. Reduced statistical power due to sample attrition and confounding by APOE 4 likely underlie this attenuation. Our findings underscore the crucial need for larger-sample size prospective cohort studies to investigate the independent association between dietary patterns and long-term cognitive health since the true effect of diet on cognition might be subtle to detect. In addition, PBF might have different impacts on different cognitive domains so findings from this study support further investigation of cognitive performance by looking at cognitive tests relevant to each cognitive domain separately. This study looked at the plant-based components in the diet, but the processing level or quality of these foods were not evaluated, which warrants further investigation into these details for a more comprehensive understanding of the impact of PBF on cognition. Despite the limitations, our findings contribute to the growing understanding of the relationship between diets and cognition in general. Future investigation should explicitly examine the potential for interaction effects between PBF consumption and genetic risk factors, such as APOE 4 allele status, to gain a more comprehensive understanding of their complex interplay in cognitive ageing. We also emphasise the importance of early interventions for age-related cognitive health diseases, especially in the higher risk group of our population—women transitioning into menopause.

## Supplementary Information

Below is the link to the electronic supplementary material.

Supplementary file1 (DOCX 846 kb)

## Data Availability

WHAP data access for this project was approved by BioGrid Australia and the data custodian in December 2022. The datasets enabling the analyses for this article are available by application to BioGrid Australia Limited (https://www.biogrid.org.au/).

## Abbreviations

AD: Alzheimer’s disease
APOE: Apolipoprotein E
BMI: Body mass index
DASH: Dietary approach to stop hypertension
DP: Dietary pattern
DQES: Dietary questionnaire for epidemiological studies
GCCS: Global cognitive composite score
MD: Mediterranean diet
MIND: Mediterranean-DASH intervention for neurodegenerative delay
PBF: Plant-based food
WHAP: Women’s Healthy Ageing Project
