# Supplementary material for: The proportion of plant-based food consumption during midlife and cognitive health in later life in Australian women: data from the Women’s Healthy Ageing Project (WHAP)
Source: Eur J Nutr. 2025 Oct 9;64(7):292. doi: 10.1007/s00394-025-03786-8 (PMC12511269; doi:10.1007/s00394-025-03786-8)
Supplement: Supplementary file 1 — Supplementary file1 (DOCX 846 kb) [file 394_2025_3786_MOESM1_ESM.docx]

**TITLE:**

**THE PROPORTION OF PLANT-BASED FOOD CONSUMPTION DURING MIDLIFE AND COGNITIVE HEALTH IN LATER LIFE IN AUSTRALIAN WOMEN – DATA FROM THE WOMEN’S HEALTHY AGEING PROJECT (WHAP)**

**Author names:**

**Phuong Le^1^, Cassandra Szoeke^2*^, Kaitlin Day^1,3^, Russell Conduit^1^, Sharayah Carter^1^, Catherine Itsiopoulos^1^**

**Affiliations:**

^1^School of Health and Biomedical Sciences, RMIT University, 124 La Trobe Street, Melbourne, VIC 3000, Australia

^2^Monash Centre for Health Research and Implementation, Monash University, Wellington Road, Clayton, VIC 3800, Australia

^3^Department of Nutritional Sciences, School of Life Course & Population Sciences, Faculty of Life Sciences and Medicine, King’s College London, United Kingdom

**Corresponding author:**

*Correspondence: Cassandra.szoeke@monash.edu

**SUPPLEMENTARY FILES**

**Supplementary Appendix 1.** Dietary questionnaire for Epidemiological Studies Version 2 (DQES v2) – Sample


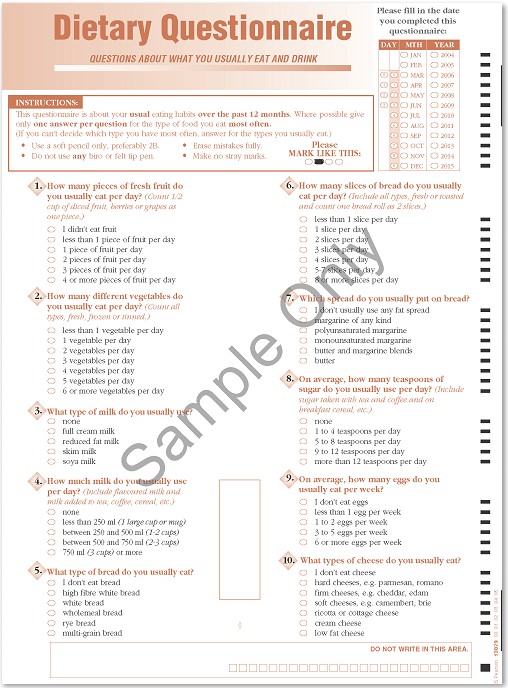


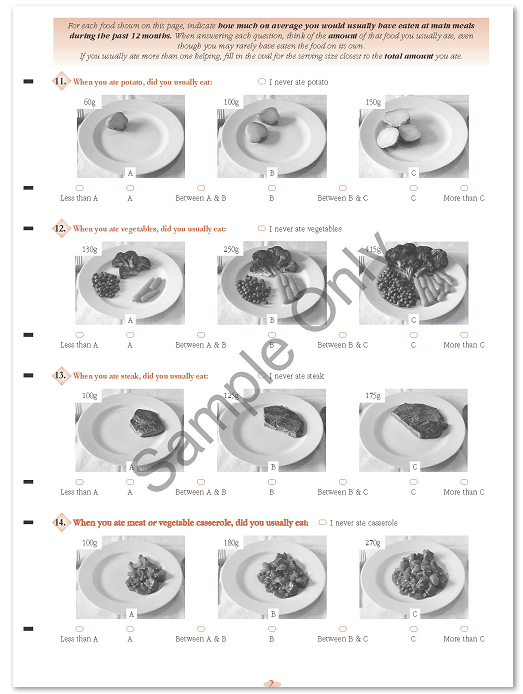


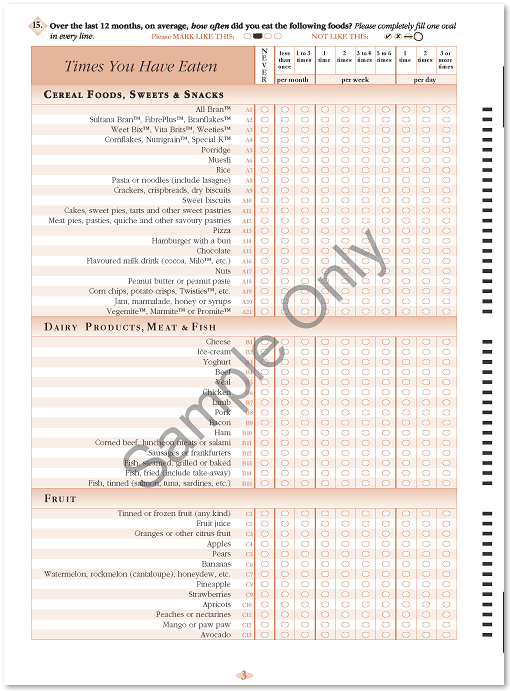


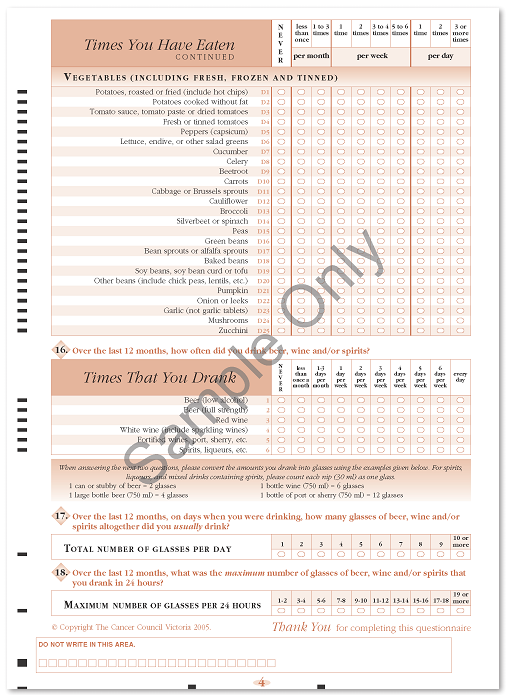


**Supplementary Appendix 2.** List of 54 plant and plant-derived food items chosen from the Food Intakes output of the questionnaire for the analyses of this study

| **Fruits** | **Vegetables** | **Other plant-based foods** |
| --- | --- | --- |
| Apples | Baked beans | High fibre white bread |
| Apricots | Beansprouts | Wholemeal bread |
| Avocado | Beetroot | Rye bread |
| Banana | Broccoli | Multigrain bread |
| Mango | Cabbage | White bread |
| Melon | Capsicum | Nuts |
| Oranges | Carrots | Allbran |
| Peaches | Cauliflower | Branflakes |
| Pears | Celery | Weetbix |
| Pineapples | Cucumber | Cornflakes |
| Strawberries | Garlic | Muesli |
| Fruit juice | Green beans | Porridge |
| Tinned fruit | Lettuce | Rice |
|  | Mushroom | Pasta |
|  | Onion | Peanut butter, jam |
|  | Other beans | Tomato sauce |
|  | Peas | Vegemite |
|  | Potato |  |
|  | Pumpkin |  |
|  | Spinach |  |
|  | Tofu |  |
|  | Tomatoes |  |
|  | Zucchini |  |

**Supplementary Table 1.** Comparison of outcome measures and covariates (measured at follow-up) of the included (completed the cognitive tests in 2012 and the dietary questionnaire in 1998) vs. the excluded participants (completed the cognitive tests in 2012 but no dietary data in 1998)

|  | **Included (n=186)** | **Excluded (n=48)** | **p value** |
| --- | --- | --- | --- |
| ***Outcome measures:*** | | | |
| **CERAD delayed recall score**  Mean (sd; range) | **4.7**  (2.5; 0 to 10) | **5.3**  (2.3; 1 to 10) | 0.14 |
| **CERAD delayed recall Z-score**  Mean (sd; range) | **-1.31**  (1.43; -5.14 to 1.81) | **-0.84**  (1.36; -4.43 to 1.24) | ***0.045*** |
| **Global cognitive composite score**  Mean (sd; range) | **-0.36**  (0.66; -3.39 to 1) | **-0.22**  (0.63; -1.55 to 1.02) | 0.18 |
| ***Covariates:*** | | | |
| **Age (yrs)**  Mean (SD; range) | **70**  (2.5; 66.1 to 77.3) | **70.8**  (3; 64.9 to 76.9) | 0.08 |
| **BMI (kg/m^2^)**  Mean (sd; range) | **27.9**  (5.4; 18.3 to 54.2)  (missing: 3) | **28.4**  (5.6; 19.1 to 50) | 0.60 |
| **Education**  < or = 12 years, n (%)  >12 years, n (%) | **105** (56.5%)  **81** (43.5%) | **32** (66.7%)  **16** (33.3%) | 0.25 |
| **Currently smoking** n (%)  (cell sample <5) | **14** (7.5%)  (missing: 3) | **3** (6.3%) | 0.78 |
| **APOE 4 carrier**  n (%) | **49** (26.3%)  (missing: 8) | **18** (37.5%)  (missing: 6) | 0.06 |
| **Physical activity (METmin/week)**  Mean (sd; range) | **4914.8**  (4849; 30 to 31404)  (missing: 9) | **5219**  (4890; 528 to 28844.6)  (missing: 4) | 0.71 |

**Supplementary Table 2.** Adjusted multiple linear regression models for the association between quartiles of Plant-based Food proportion in daily diet (1998) and Global Cognitive Composite Score (2012) in APOE 4(+) group

| **Model** | **Variables** | **B** | **95% CI lower** | **95% CI upper** | **p-value** |
| --- | --- | --- | --- | --- | --- |
| **Model 1,**  **N = 49** | **PBF proportion quartiles** | | | | |
|  | Quartile 1 | -reference- | | | |
|  | Quartile 2 | 0.54 | -0.24 | 1.31 | 0.17 |
|  | Quartile 3 | 0.67 | 0.03 | 1.32 | ***0.04*** |
|  | Quartile 4 | 0.57 | -0.16 | 1.29 | 0.12 |
|  | **Covariates** | | | | |
|  | Age (years) | -0.02 | -0.12 | 0.08 | 0.68 |
|  | Education (≥ vs. < 12 years) | 0.29 | -0.23 | 0.8 | 0.27 |
|  | **Model’s adjusted R^2^ (p-value)** | 0.096 (0.095) | | | |
| **Model 2,**  **N = 44** | **PBF proportion quartiles** | | | | |
|  | Quartile 1 | -reference- | | | |
|  | Quartile 2 | 0.18 | -0.53 | 0.9 | 0.6 |
|  | Quartile 3 | 0.43 | -0.18 | 1.04 | 0.16 |
|  | Quartile 4 | 0.42 | -0.25 | 1.08 | 0.21 |
|  | **Covariates** | | | | |
|  | Age (years) | 0.01 | -0.09 | 0.11 | 0.83 |
|  | Education (≥ vs. < 12 years) | 0.35 | -0.13 | 0.84 | 0.15 |
|  | Energy intake (kJ/day) | -9.76e-6 | 0.0 | 0.0 | 0.84 |
|  | Physical activity  (METmin/week) | 9.75e-5 | 0.0 | 0.0 | ***0.01*** |
|  | **Model’s adjusted R^2^ (p-value)** | 0.156 (0.07) | | | |
| **Model 3,**  **N = 47** | **PBF proportion quartiles** | | | | |
|  | Quartile 1 | -reference- | | | |
|  | Quartile 2 | 0.52 | -0.36 | 1.39 | 0.24 |
|  | Quartile 3 | 0.69 | -0.04 | 1.42 | 0.07 |
|  | Quartile 4 | 0.52 | -0.3 | 1.33 | 0.21 |
|  | **Covariates** | | | | |
|  | Age (years) | -0.03 | -0.14 | 0.08 | 0.55 |
|  | Education (≥ vs. < 12 years) | 0.32 | -0.24 | 0.87 | 0.26 |
|  | Energy intake (kJ/day) | 5.85e-7 | 0.0 | 0.0 | 0.99 |
|  | BMI (kg/m^2^) | 0.02 | -0.04 | 0.07 | 0.52 |
|  | **Model’s adjusted R^2^ (p-value)** | 0.048 (0.26) | | | |

**Supplementary Table 3.** Change in PBF proportion from 1998 – 2012 of the participants by quartiles of PBF proportion in 1998 (N=167)

| **Quartile of PBF proportion in daily in 1998** | **Quartile 1** | **Quartile 2** | **Quartile 3** | **Quartile 4** |  |
| --- | --- | --- | --- | --- | --- |
| **Mean change in the PBF proportion in daily diet from 1998 – 2012 (%)**  Mean (sd; range) | **1.54%**  (10.9%;  -18.7% to 25.5%) | **-1.49%**  (10%;  -37.2% to 29.3%) | **-4.9%**  (8.8%;  -27.8% to 17.6%) | **-13.4%**  (9.6%;  -36.2% to 8.7%) | p <0.001 |
| **Number of participants who increased PBF proportion:** n (%) | **21 (45.7%)** | **19 (40.4%)** | **11 (23.4%)** | **4 (8.7%)** | Total: 55 (32.9%) |
| **Number of participants who decreased PBF proportion**: n (%) | **22 (47.8%)** | **23 (48.9%)** | **30 (63.8%)** | **37 (80.4%)** | Total: 112 (67.1%) |

*Note*: The change in PBF intake was calculated by subtracting the PBF proportion in 2012 by that in 1998

**Supplementary Table 4.** Simple linear regression models for the association between changes of Plant-based Food proportion in daily diet from midlife (1998) to late-life (2012) and late-life Global Cognitive Composite Score (2012)

| **Predictor** | | **B** | **CI Lower^a^** | **CI Upper^a^** | **p-value** |
| --- | --- | --- | --- | --- | --- |
| Change in % of PBF in daily diet from 1998 to 2012 | Model 1^b^ | -0.01 | -0.82 | 0.8 | 0.981 |
|  | Model 2^c^ | -0.054 | -0.77 | 0.67 | 0.883 |
| ^a^: Lower and upper bounds of 95% confidence interval for B value  ^b^: Model 1 includes all participants who had available data for this analysis, ***N = 167***, ΔR^2^ = -0.006, p = 0.981  ^c^: Model 2 excludes 7 outliers identified using Casewise Diagnostics, ***N = 160***, ΔR^2^ = -0.006, p = 0.883 | | | | | |
